# Current Iodine Nutrition Status and Awareness of Iodine Deficiency in Tuguegarao, Philippines

**DOI:** 10.1155/2014/210528

**Published:** 2014-10-13

**Authors:** Bu Kyung Kim, Jee-Yeong Jeong, Kwang-Hyuk Seok, Andrew S. Lee, Chul Ho Oak, Ghi Chan Kim, Chae-Kyeong Jeong, Sung In Choi, Pablo M. Afidchao, Young Sik Choi

**Affiliations:** ^1^Department of Internal Medicine, Kosin University College of Medicine, 262 Gamcheon-ro, Seo-gu, Busan 602-703, Republic of Korea; ^2^Department of Biochemistry, Kosin University College of Medicine, Busan 602-703, Republic of Korea; ^3^Cancer Research Institute, Kosin University College of Medicine, Busan 602-703, Republic of Korea; ^4^Department of Microbiology, Kosin University College of Medicine, Busan 602-703, Republic of Korea; ^5^Institute for International Healthcare Cooperation, Kosin University College of Medicine, Busan 602-703, Republic of Korea; ^6^Department of Physical Medicine and Rehabilitation, Kosin University College of Medicine, Busan 602-703, Republic of Korea; ^7^Department of Chemistry, Boston College, Chestnut Hill, MA 02467, USA; ^8^Department of Food Science and Nutrition, Pusan National University, Busan 609-838, Republic of Korea; ^9^Department of Pharmacology and Biochemistry, Cagayan State University, College of Medicine, 3500 Tuguegarao, Philippines

## Abstract

The Philippines is one of the countries where adequate iodine status has been achieved. However, iodine deficiency still remains an important public health problem in this country. In this study, we evaluated iodine nutrition status and investigated an awareness status of iodine deficiency targeting high school students of Tuguegarao, Philippines. A total of 260 students provided samples for urinary iodine analysis, among which 146 students completed thyroid volume measurement by ultrasonography and answering the questionnaires. The median urinary iodine level was 355.3 *µ*g/L and only 3.8% of the students were in the range of iodine deficiency status according to the ICCIDD criteria. Although 62.3% of students answered that they can list problems resulting from iodine deficiency, a majority of students (70.5%) were unable to identify problems other than goiter. They did not appreciate that adequate iodine levels are important during pregnancy and for development of children. 33.6% of students answered that they did not use iodized salt and the biggest reason was that they did not find it necessary. Based on these results, we suggest that a future strategy should be focused on vulnerable groups to completely eliminate iodine deficiency, including women at their reproductive ages and during pregnancy.

## 1. Background

Over the past two decades, global iodine nutrition status has markedly been improved. According to the International Council for Control of Iodine Deficiency Disorders (ICCIDD), the number of iodine deficient countries was decreased from 54 countries in 2003 to only 32 countries in 2011 [1–4]. However, iodine deficiency still remains one of the most important public health problems. Iodine deficiency causes multiple health problems including endemic goiter, cretinism, intellectual impairments, growth retardation, neonatal hypothyroidism, and increased pregnancy loss and infant mortality [5]. Iodine is a key nutrient in the synthesis of thyroid hormones which take pivotal roles particularly in fetal neurodevelopment in uterus [6]. Recently, several studies reported that even mild iodine deficiency during gestation may lead to damage of fetal brain development, a decrease in intelligence quotient (IQ), and impairment of cognitive functions in school-age children [4, 7–9]. Therefore, iodine deficiency has been getting renewed attention even in areas of mild iodine deficiency.

The Philippines has been overcoming iodine deficiency through government policies such as ASIN law which mandated the addition of iodine to all salt for animal and human consumption since 1995 [10]. As a result of these efforts, the Philippines is currently classified as adequate iodine nutrition area [1, 4]. According to the 2003 national study in the Philippines, 6–12-year-old children's median urinary iodine (UI) was 201 *μ*g/L and the percentage of children with severe iodine deficiency (UI < 50 *μ*g/L) was 11.4% [11]. In our previous study [12], we reported the median UI of 80 school children (also aged 6–12 years old) to be 279 *μ*g/L (ranged between 20.8 and 874 *μ*g/L), and 12.4% had insufficient iodine (UI < 100 *μ*g/L). Despite this improvement, endemic goiter has still been recognized in most parts of the country and 50,000 Filipino newborns have mental problems due to iodine deficiency, according to the news article issued by ICCIDD on May 7, 2013. Therefore, it is necessary to find more specific and focused strategies on vulnerable groups of iodine deficiency to eradicate related disorders even in adequate iodine nutrition area.

In this study, we examined urinary iodine concentrations and thyroid volumes to evaluate iodine nutrition status among high school students in Tuguegarao, Philippines. We also investigated an awareness status of iodine deficiency using questionnaires to identify the areas we need to further focus on, in order to eliminate iodine deficiency in the country.

## 2. Methods

### 2.1. Study Population

This study was conducted on high school students in Tuguegarao, Cagayan Valley, Philippines, in July 2013. A total of 260 high school students provided samples for urinary iodine analysis, among which 146 students (median age = 15, range 12–25) completed measuring thyroid volume by ultrasonography and answering questionnaires for awareness of iodine deficiency. Among them, 79 (54.1%) were girls and 67 (38.4%) were boys.

This study was approved by the Institutional Review Board (IRB) of Kosin University College of Medicine. The study was also approved by the mayor of Tuguegarao and Cagayan State University, Cagayan, Philippines. Informed consent was obtained from the subjects. Height (cm) and weight (kg) were measured for all the participants, and body surface area (BSA) was calculated by the following formula [13]:

(1) $$\begin{array}{c} \text{BSA}=\text{Weight}\;{\left(\text{kg}\right)}^{0.425}\times \text{Height}\;{\left(\text{cm}\right)}^{0.725}\times 71.84\times 10^{-4}. \end{array}$$

### 2.2. Measurement of Urinary Iodine Concentrations

The spot urine samples (4.0 to 11.5 mL) were collected from 260 students in wide-mouthed screw capped plastic bottles. The samples were immediately filtered and refrigerated until analysis. Urinary iodine (UI) concentrations were determined by modified microplate method employing ammonium persulfate digestion followed by Sandell-Kolthoff reaction [14, 15], which is one of the three most common methods used by participants of Ensuring the Quality of Iodine Procedures (EQUIP) of the US Centers for Disease Control and Prevention (CDC) [16]. Briefly, all urine samples and standards (0, 20, 40, 80, 120, 200, and 300 *μ*g/L KIO_3_) were allowed to reach ambient temperature; then 250 *μ*L of each sample and standard solutions were mixed with 1 mL of ammonium persulfate solution. The mixtures were incubated on a heating block for 60 min at 95°C and cooled to room temperature. After transferring 50 *μ*L of each mixture to a 96-well plate in duplicate, 100 *μ*L of arsenious acid solution and 50 *μ*L ceric ammonium sulfate solution were added sequentially and incubated for 30 seconds and 30 min, respectively. After reading the absorbance at 405 nm, UI concentrations were determined using standard curves constructed for each plate. We also tested Standard Reference Material (SRM) 2670a (kindly provided by the US CDC) and confirmed the accuracy of our assays. The iodine deficiency grade was defined according to the WHO's median UI level criteria as follows: <20 *μ*g/L: severe iodine deficiency, 20–49 *μ*g/L: moderate iodine deficiency, 50–99 *μ*g/L: mild iodine deficiency, 100–199 *μ*g/L: optimal, 200–299 *μ*g/L: more than adequate, and >300 *μ*g/L: excessive [17]. The creatinine concentrations were measured with an ACCESS 2 produced by Beckman Coulter and an LX20 produced by Beckman Coulter (Brea, CA, USA) for each sample.

### 2.3. Measurement of Thyroid Volumes

Thyroid volumes were measured using a portable ultrasound instrument equipped with a 10 MHz linear transducer (LOGIQ BOOK XP, GE healthcare, Seoul, Republic of Korea). The examination was performed by a single experienced endocrinologist with two additional persons assisting and record-keeping. The subjects were in the supine position with the hyperextension of neck for examination. The volume of each lobe was calculated using the following formula [18]: width (cm) × length (cm) × depth (cm) × 0.479. The thyroid volume was then the sum of the volumes of both lobes. Isthmus volume was not taken into account.

### 2.4. Questionnaires

The questionnaires consisted of five main questions and two subquestions: (1) have you heard about the problems associated with iodine deficiency in the environment and insufficient intake of iodine in the human body?; (2) can you list some problems (disorders) resulting from iodine deficiency?; (2-1) which problems (disorders)? (please select all you can list); (3) where do you receive information about iodine deficiency disorders and preventing them?; (4) what do you need to do about iodine deficiency?; (5) do you buy iodized salt?; (5-1) (if you answered “no” to question (5) what is (are) the reason(s) you do not buy iodized salt?

### 2.5. Statistical Analysis

For each subject, the observed measurements were carried out by an experienced endocrinologist. The thyroid volume was represented by the value of minimum, maximum, median, and mean ± standard deviation according to age. We compared urine iodine levels with the previous National Nutritional Survey (NNS) data of 1998 and 2003 by descriptive statistics. Between the two groups who use iodized salt and do not use iodized salt, urinary iodine, thyroid volume, height, and weight were compared by dependent *t*-test. Statistical analysis was performed using the Statistical Package for Social Sciences (SPSS, version 20.0, Chicago, IL, USA).

## 3. Results

### 3.1. Urinary Iodine

**Figure 1:**
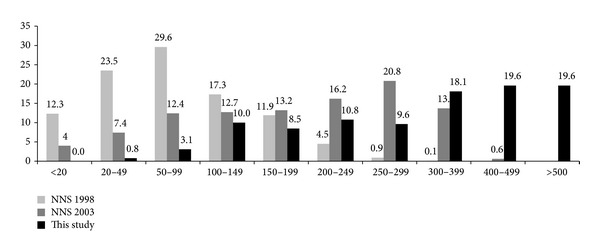
Distribution of urinary iodine concentrations (*μ*g/L) in schoolchildren in the Philippines in 1998, 2003, and 2013.

A median urinary iodine level was 355.3 *μ*g/L and creatinine adjusted level was 279.4 *μ*g/g. According to the ICCIDD criteria, only 3.8% of students were in the iodine deficiency status. After being adjusted with creatinine, the proportion of iodine deficiency was decreased to 1.5% (Table 1). There was no significant difference of UI between girls and boys (398.54 ± 230.0 versus 343.94 ± 191.7,  *P* = 0.136). In 1998, 65.4% of the Philippines population had iodine deficiency (mean UI level < 100 *μ*g/L), and this was decreased to 23.8% in 2003 (Figure 1).

### 3.2. Thyroid Volume

As age increased, students' height, weight, BSA, and thyroid volume also increased as expected. However, the student group at the age of 19 or older showed smaller height, weight, and BSA, but larger thyroid volume than the student group at the age of 18 (Table 2).

### 3.3. Questionnaires

**Figure 2:**
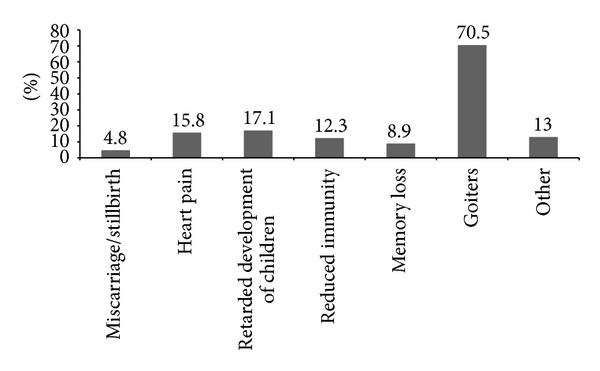
Problems list that the students chose as a result of iodine deficiency.

**Figure 3:**
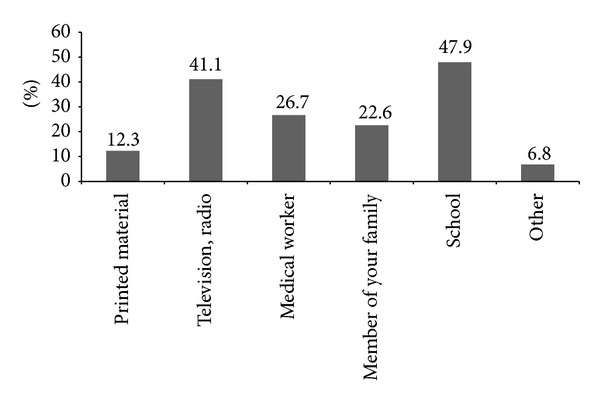
Routes of getting information about iodine deficiency disorders and prevention of them.

**Figure 4:**
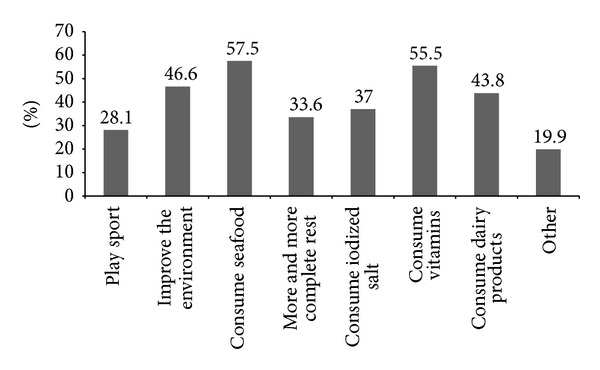
Preventing methods of iodine deficiency with the students listed.

**Figure 5:**
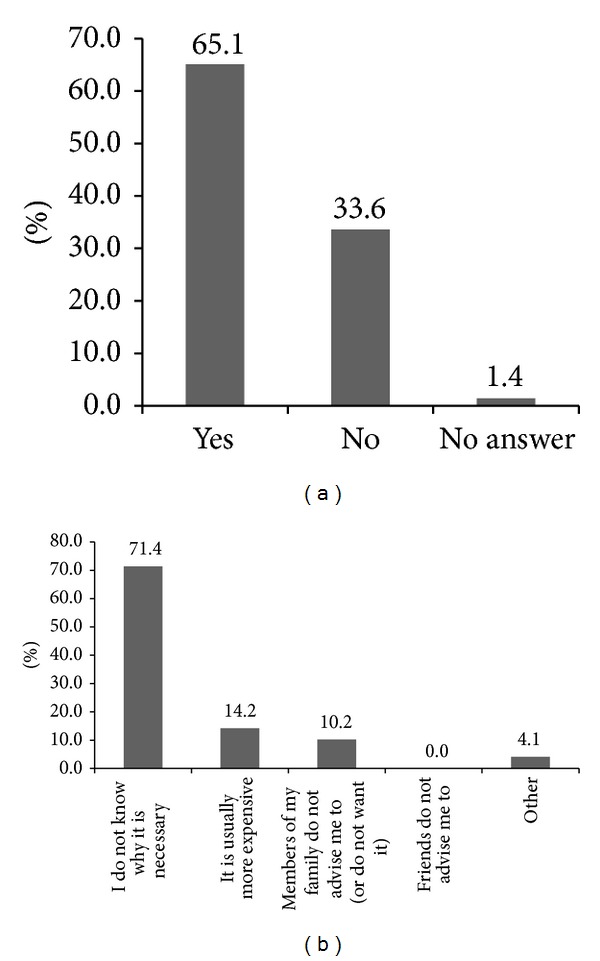
Using status of iodized salt (a) and the reasons of not using iodized salt (b).

For the first question, 56.8% of the students answered that they have heard about the problems associated with iodine deficiency. Although 62.3% of students answered that they can list some problems (disorders) resulting from iodine deficiency, most students (70.5%) chose goiter as the only problem (Figure 2). The main sources from which students received information about iodine deficiency disorders were school (47.9%), followed by television and radio (41.1%) as shown in Figure 3. To the question on how to improve iodine deficiency, 57.5% of students answered that they need to consume seafood and only 37% answered that they need to consume iodized salt (Figure 4). More than half of students (65.1%) answered that they use iodized salt, but 33.6% answered that they do not use iodized salt (Figure 5(a)). The biggest reason in not using iodized salt was that they do not know why it is needed (Figure 5(b)).

### 3.4. Urinary Iodine and Thyroid Volume according to Consuming Ionized Salt

There was no significant difference in urinary iodine levels, height, and weight between the iodized salt using group and nonusing group. However, the thyroid volume was larger in the latter (4.35 ± 2.47 versus 3.71 ± 1.03,  *P* = 0.032) (Table 3).

## 4. Discussion

WHO defined iodine deficiency as “the single most important preventable cause of brain damage” worldwide [19]. Fortunately, iodine deficiency is one of the simplest and least expensive nutrient problems, which can be prevented through salt iodization. The annual costs of iodized salt are estimated to be only 0.2–0.5$ per child [3, 4, 19]. The global effort to control iodine deficiency has achieved remarkable success especially over the last two decades. Before 1990, only a few countries were in the iodine sufficient status. In 1990, at the United Nations World Summit for Children and World Health Assembly, world leaders established the goal of eliminating iodine deficiency worldwide [20]. From 2003 to 2011, the number of iodine sufficient countries increased from 67 to 111 [21]. Currently, more than 70% of all households worldwide have access to iodized salt, being markedly improved from less than 10% in 1990 [22, 23].

The Philippines also turns out to be one of the countries with adequate iodine nutrition according to the current study, which demonstrated that the median urinary iodine level was 355.3 *μ*g/L (Table 1). In 1998, 65.4% had iodine deficiency; the percentages of severe (<20 *μ*g/L), moderate (<50 *μ*g/L), and mild (<100 *μ*g/L) deficiencies were 12.3%, 23.5%, and 29.6%, respectively. In 2003, the portion of iodine deficiency greatly decreased to 23.8%. In this study, only 3.8% was in the range of iodine deficiency (Figure 1), indicating that the Philippines has successfully achieved sufficient levels of iodine within only several years.

According to the WHO criteria, the median UI level (355.3 *μ*g/L) of this study indicates rather an excessive iodine status. The following two questions should be considered. First, why is the median UI level of this study so high? and second, does this study represent all the regions of the Philippines? If an individual consumes 5 g of salt iodized at 30 ppm, he or she gets 150 *μ*g iodine from iodized salt alone [24]. The result of UI level higher than 150 *μ*g/L suggests the possibility of other dietary sources of iodine. As we indicated in our previous study, Tuguegarao is one of the biggest cities in the Cagayan valley [12]. Therefore, there is a possibility that participants of this study are in higher socioeconomic status than the general population of the Philippines. They might consume daily foods containing more iodine and take nutritional supplements including iodine. Geographical characteristics should also be considered. Tuguegarao is not far from the ocean; therefore, higher consumption of sea food might be another possible explanation for the high levels of UI. Therefore, a larger-scale survey which includes questions regarding daily food intake should be conducted to take geographical characteristics and socioeconomic status into consideration.

In this study, we presented questionnaires to the subjects to evaluate an awareness status of iodine deficiency. Surprisingly, despite the fact that iodine deficiency status was improved, many students did not have proper knowledge regarding the condition. Most importantly, a majority of the students were unable to list the problems associated with iodine deficiency except goiter. Only 4.8% of students listed miscarriage/stillbirth and 17.1% listed retarded development of children as such problems (Figure 2). Recently, iodine deficiency is reemerging even in industrialized countries because even mild iodine deficiency in pregnant women could have an adverse effect on their children's cognitive functions [7, 8]. Moreover, pregnant women in a mild iodine deficient country exhibited high prevalence of thyroid disorder particularly in the first trimester [25]. The WHO recommended an iodine intake of 250 *μ*g per day and defined iodine deficiency as when median UIC is less than 150 *μ*g/L during pregnancy [19]. In the United Kingdom, children of pregnant women with UIC < 150 *μ*g/L had lower scores for verbal intelligence quotient (IQ), reading accuracy, and reading comprehension than those of mothers with 150 *μ*g/L or more [19]. It is established that only mild iodine deficiency in pregnancy may lead to elevated thyroid stimulating hormone (TSH) levels in fetuses and these conditions are related to cognitive and psychomotor deficits in children [9]. Several studies also suggested that iodine supplements could decrease the risk of mental developmental delay [26–28]. During lactation and the first three postnatal years, an enough supply of iodine is very important for development of children [29]. Neonatal iodine deficiency can cause goiter and also lead to moderate to severe hypothyroidism [5]. Therefore, Delange suggested that the adequate iodine dosage is 225–350 *μ*g/day during lactation and 90 *μ*g/day during the neonatal period [30]. For some children who are not breast-fed, the iodide content of synthetic milk should be controlled. Our study showed that most students did not know the importance of iodine deficiency for pregnant women. This lack of understanding may potentially lead to iodine deficiency risks during pregnancy and breast-feeding period in female students in the future.

In 1993, the WHO reaffirmed salt iodization as the central strategy to eliminate iodine deficiency [31]. Surprisingly, only 37% of students responded with the consumption of iodized salt as a way to prevent iodine deficiency (Figure 4), indicating that many students are yet to recognize the importance of iodized salt, although the Philippines government mandated salt manufacturers to sell only iodized salt through the enactment of the ASIN law in 1995 [10]. The percentage of students who answered “no” to the question on whether or not to buy iodized salt was 33.6%, among which 71.4% answered that they do not buy iodized salt because they do not know of its necessity (Figure 5). Despite these answers, the urinary iodine levels of these students were not in the range of iodine deficiency (Table 3). Therefore, we postulate that, with the enactment of the ASIN law, most Filipinos have consumed iodized salt regardless whether they recognize its importance or not. In fact, the 2008 Philippines' National Nutritional Survey (NNS) found that over 80% of commercially sold table salt is iodized, although approximately 75% contained iodine less than the WHO minimum recommendation of 15 ppm.

There was no difference in the urinary iodine levels between the group of students who answered “no” and that of students who answered “yes” to the question on whether to buy iodized salt. However, it is intriguing that the thyroid volumes were larger in the “no” group than in the “yes” group with a statistical significance (Table 3). It is possible that the students who do not appreciate the importance of iodized salt might have been exposed to iodine deficiency previously when they were younger, likely due to poor education and/or a low socioeconomic status. Follow-up studies will be necessary to investigate if these students have a higher risk of goiter development in the future and to understand how iodine sufficiency is required at younger age. The results also suggest the specific group of people who should be targeted to ameliorate iodine deficiency and its problems.

Our current study revealed that only 4% of high school students are iodine deficient in Tuguegarao, the Philippines, indicating that iodine deficiency in this age group has been nearly overcome by efforts of the Philippines government and the WHO ICCIDD. Moreover, the Philippines Food and Drug Administration ordered salt manufacturers to increase the mandatory salt iodine content from 20 ppm to 30 ppm in 2013. For this reason, even further improvement is expected [32].

In order to completely eliminate iodine deficiency, the future strategy should be more focused on vulnerable groups through more effective education. According to our survey, most students gathered information regarding iodine deficiency in school and through media (Figure 3). In addition to school-based education, sources of media should be utilized more actively to inform that (1) iodine deficiency causes much more problems beyond goiters; (2) iodine deficiency can be prevented by the consumption of iodized salt; (3) more iodine is required during pregnancy as iodine deficiency inhibits fetus growth and development. Also, nutrition surveys should be focused on vulnerable regions and population with poor education and a lower socioeconomic status to eliminate iodine deficiency effectively and completely in the Philippines.

Our study is conducted in only one area of the Philippines; however, it could be helpful for many countries with a similar iodine status around the world to establish their own public health strategies to eliminate iodine deficiency. Several studies suggested that public education programs are needed to improve iodine status [33, 34]. In addition to the public education programs, our results strongly suggest that a future strategy should be focused on vulnerable groups, including women at their reproductive ages and during pregnancy and breast-feeding period, especially in the areas of lower socioeconomic status, to completely eliminate iodine deficiency and the associated disorders.

## Figures and Tables

**Table 1:** Urinary iodine concentration from a total of 260 high school students in Tuguegarao.

	Median	Mean	95% confidence interval of mean	Maximum	Minimum	Number of iodine deficiency (%) <100 *μ*g/L
Urinary iodine (*μ*g/L)	355.3	378.5	352.5–407.9	1402.69	29.27	10 (3.8%)
Cr adjusted iodine (*μ*g/g)	279.4	311.96	293.5–332.8	910.31	27.23	4 (1.5%)

**Table 2:** Height, weight, and thyroid volume according to age.

Age	Height (cm)	Weight (Kg)	BSA (m^2^)	Thyroid volume
≤13 (*n* = 12)	152.7 ± 6.72	40.7 ± 6.31	1.31 ± 0.126	3.09 ± 0.86
14 (*n* = 50)	153.1 ± 5.83	44.8 ± 7.11	1.38 ± 0.121	3.78 ± 1.12
15 (*n* = 54)	157.00 ± 5.99	48.4 ± 5.83	1.45 ± 0.117	3.94 ± 1.24
16 (*n* = 20)	157.30 ± 5.76	49.5 ± 6.00	1.47 ± 0.106	3.77 ± 1.20
17 (*n* = 2)	159.50 ± 4.95	50.5 ± 3.53	1.50 ± 0.076	4.55 ± 1.02
18 (*n* = 4)	160.75 ± 5.06	53.8 ± 4.79	1.55 ± 0.067	3.56 ± 1.16
≥19 (*n* = 4)	156.75 ± 4.03	49.5 ± 5.45	1.47 ± 0.86	4.35 ± 0.65

**Table 3:** Urinary iodine and thyroid volume according to consuming ionized salt.

	Using iodized salt	Nonusing iodized salt	*P* value
Urinary iodine	394.71 ± 237.65	336.88 ± 172.35	0.099
Thyroid volume	3.71 ± 1.03	4.35 ± 2.47	0.032
Height (cm)	155.15 ± 6.13	156.35 ± 6.27	0.277
Weight (Kg)	46.61 ± 6.49	47.69 ± 7.33	0.384
